# Quality Assessment of the Chinese Clinical Trial Protocols Regarding Treatments for Coronavirus Disease 2019

**DOI:** 10.3389/fphar.2020.01330

**Published:** 2020-08-27

**Authors:** Jiaxing Zhang, Yiling Lu, Joey Sum-wing Kwong, Xiaosi Li, Wenyi Zheng, Rui He

**Affiliations:** ^1^ Department of Pharmacy, Guizhou Provincial People’s Hospital, Guiyang, China; ^2^ Department of Pharmacy, The Third People’s Hospital of Chengdu, Chengdu, China; ^3^ Global Health Nursing, Graduate School of Nursing Science, St. Luke’s International University, Tokyo, Japan; ^4^ Department of Pharmacy, Hospital of Chengdu Office of People’s Government of Tibetan Autonomous Region, Chengdu, China; ^5^ Experimental Cancer Medicine, Department of Laboratory Medicine, Karolinska Institute, Stockholm, Sweden; ^6^ Clinical Research Center and Center of Allogeneic Stem Cell Transplantation(CAST), Karolinska University Hospital Huddinge, Stockholm, Sweden

**Keywords:** 2019 Novel Coronavirus, coronavirus disease 2019, therapies, randomized controlled trial protocol, cross-sectional analysis

## Abstract

**Background:**

With the global spread of coronavirus disease 2019 (COVID-19), an increasing number of clinical trials are being designed and executed to evaluate the efficacy and safety of various therapies for COVID-19. We conducted this survey to assess the methodological quality of registry protocols on potential treatments for COVID-19.

**Methods:**

Clinical trial protocols were identified on the ClinicalTrials.gov and the Chinese Clinical Trial Registry. Protocols were screened by two investigators independently against pre-defined eligibility criteria. Quality of the included protocols was assessed according to the modified 14-item SPIRIT (Standard Protocol Items: Recommendations for Interventional Trials) 2013 Statement.

**Results:**

We included 82 randomized controlled trial (RCT) protocols investigating treatment modalities for COVID-19. These ongoing trials are being conducted in 16 provinces, autonomous regions, and municipalities of China, and study interventions were either Western medicines (n = 56) or traditional Chinese medicine (n = 26). Findings of our quality assessment indicated that the existing trial protocols could be further improved on several aspects, including selection and definition of outcome measures, descriptions of study interventions and comparators, study subject recruitment time, definition of study inclusion and exclusion criteria, and allocation concealment methods. Descriptions of random sequence generation methodologies were accurate for the majority of included trial protocols (n = 64; 78.05%); however, reporting of allocation concealment remained unclear in 63 (76.83%) protocols. Therefore, the overall risk of selection bias across these RCTs was judged to be unclear. A total of 52 (63.41%) included RCT protocols were open-label trials and are thus associated with a high risk of performance bias and detection bias.

**Conclusion:**

Quality of currently available RCT protocols on the treatments for COVID-19 could be further improved. For transparency and effective knowledge translation in real-world clinically settings, it is important for trial investigators to standardize baseline treatments for patients with COVID-19 and assess clinically important core outcome measures. Despite eager anticipation from the public on the results of effectiveness trials in COVID-19, robust design, execution, and reporting of these trials should be regarded as high priority.

## Introduction

In December 2019, coronavirus disease 2019 (COVID-19), an infectious disease caused by severe acute respiratory syndrome coronavirus 2 (SARS-CoV-2), outbroke in Wuhan, Hubei province, China (Hui et al., 2020; Lu et al., 2020). As of March 4, 2020, a total of 80,409 cases have been confirmed and over 3,000 deaths were reported in China alone (National Health Commission of the People’s Republic of China, 2020). The total numbers of confirmed cases and deaths in other countries were 12,668 and 214, respectively (World Health Organization, 2020a). The World Health Organization (WHO) declared COVID-19 a public health emergency of international concern (World Health Organization, 2020b).

There is a paucity of evidence regarding the therapeutic options for COVID-19. Four case series involving 41, 99, 138, and 1099 patients with laboratory-confirmed COVID-19, respectively, have been published (Chen N. et al., 2020; Guan et al., 2020; Huang et al., 2020; Wang D. et al., 2020); and a wide array of antiviral therapies such as oseltamivir, ganciclovir, and lopinavir/ritonavir were used. However, the efficacy of these drugs was not evaluated. Two preclinical studies showed that remdesivir, chloroquine, arbidol, and darunavir could effectively inhibit SARS-CoV-2 (Huang, 2020; Wang M. et al., 2020); and two clinical studies investigated the effects of remdesivir, arbidol, lopinavir/ritonavir, and Shufeng Jiedu capsules in treating COVID-19 (Holshue et al., 2020; Wang Z. et al., 2020). Driven by the effectiveness of lopinavir/ritonavir in the early treatment of patients with Severe Acute Respiratory Syndrome (SARS), a systematic review suggested that it could also serve as an experimental antiviral therapy for CoVID-19, in particular for newly diagnosed patients (Jiang et al., 2020). Nevertheless, a retrospective cohort study of 134 patients did not find any effects of lopinavir/ritonavir and arbidol on relieving symptoms or accelerating virus clearance among patients with COVID-19 (Chen J. et al., 2020). The latest Guidance for Corona Virus Disease 2019 Prevention, Control, Diagnosis and Management (GCVD2019PCDM) guidelines proposed alpha-interferon nebulization, lopinavir/ritonavir, ribavirin, chloroquine phosphate, and arbidol as antiviral treatments (National Health Commission of PRC and National Administration of Traditional Chinese Medicine of the RPC, 2020).

Randomized controlled trial (RCT) by design is the gold standard for evaluating the effects of interventions. Up to now, more than 80 clinical trials exploring potential treatment options for COVID-19 are registered/ongoing in China (Maxmen, 2020). For research transparency and validity, clinical trials should be pre-registered in a validated study register where study plans and protocols are available in the public domain (Chhapola et al., 2018; Hendarto et al., 2019). To our knowledge, there is currently no attempt to assess the methodological quality of existing trial protocols in the field of COVID-19 and we thus conducted this cross-sectional analysis to evaluate the quality of clinical trial protocols on potential COVID-19 treatments.

## Materials and Methods

### Study Design

This is a cross-sectional analysis of clinical trial protocols on treatment modalities of COVID-19.

### Data Source

Clinical trial protocols were searched on ClinicalTrials.gov with the terms “2019-nCov” or “Novel Coronavirus” or “COVID-19” or “SARS-Cov-2” from its earliest records to February 18, 2020. The Chinese Clinical Trial Registry (CCTR) (http://www.chictr.org.cn/index.aspx) was also searched with Chinese terms.

### Eligibility Criteria

We included intervention trial protocols meeting the following criteria: (1) RCT by design; (2) study participants with laboratory-confirmed CoVID-19; (3) involving Western medicine (WM) or traditional Chinese medicine (TCM) as a treatment intervention. Study protocols enrolling patients treated in the recovery phase were excluded.

### Study Selection and Data Extraction

Two investigators independently screened the protocols for inclusion and assessed their quality against pre-defined inclusion and exclusion criteria. Any disagreement in the process of study selection was resolved by discussion. Two authors independently extracted the following data from included protocols: (1) basic information: registry number, title, primary sponsor, location, institutional level, study execution time, source of funding; (2) population information: inclusion criteria, exclusion criteria, age, and sample size; (3) interventions: medicine, dosage, usage, course of treatment, and number of study groups; (4) outcomes: definition, time-point of measurement, and method of measurement for primary and secondary outcomes; (5) study design: study type, randomization procedure, allocation concealment, blinding, data collection and management, ethical permit, and informed consensus.

### Quality Assessment

Two investigators independently appraised the quality of each included protocols using the modified Standard Protocol Items: Recommendations for Interventional Trials (SPIRIT) 2013 Statement, and any disagreements were resolved by discussion. The modified SPIRIT 2013 was developed following the SPIRIT 2013 Statement (Chan et al., 2013) and the information provided on the ClinicalTrial.gov and the Chinese Clinical Trial Registry. In the modified SPIRIT 2013 checklist, the evaluation items include (1) specific objectives or hypotheses; (2) conflict of interest; (3) clear enrolment schedule; (4) specific participant inclusion and exclusion criteria; (5) sufficient details about interventions for each group, including how and when interventions are applied; (6) matching between grouping and the research purpose; (7) sufficient details about outcome measurement; (8) suitability of the primary outcome; (9) all the collaborating institutions listed in a multicenter study; (10) randomization sequence generation; (11) allocation concealment; (12) blinding; (13) data collection and management methods; (14) ethical permit. We categorized the judgments as low, high, or unclear risk of bias.

### Statistical Synthesis

Statistical analysis was conducted using SPSS (version19.0) software. The rate or constituent ratio was used to describe qualitative data.

## Results

### Search Result and Baseline Characteristics of Included Trial Protocols

**Figure 1:**
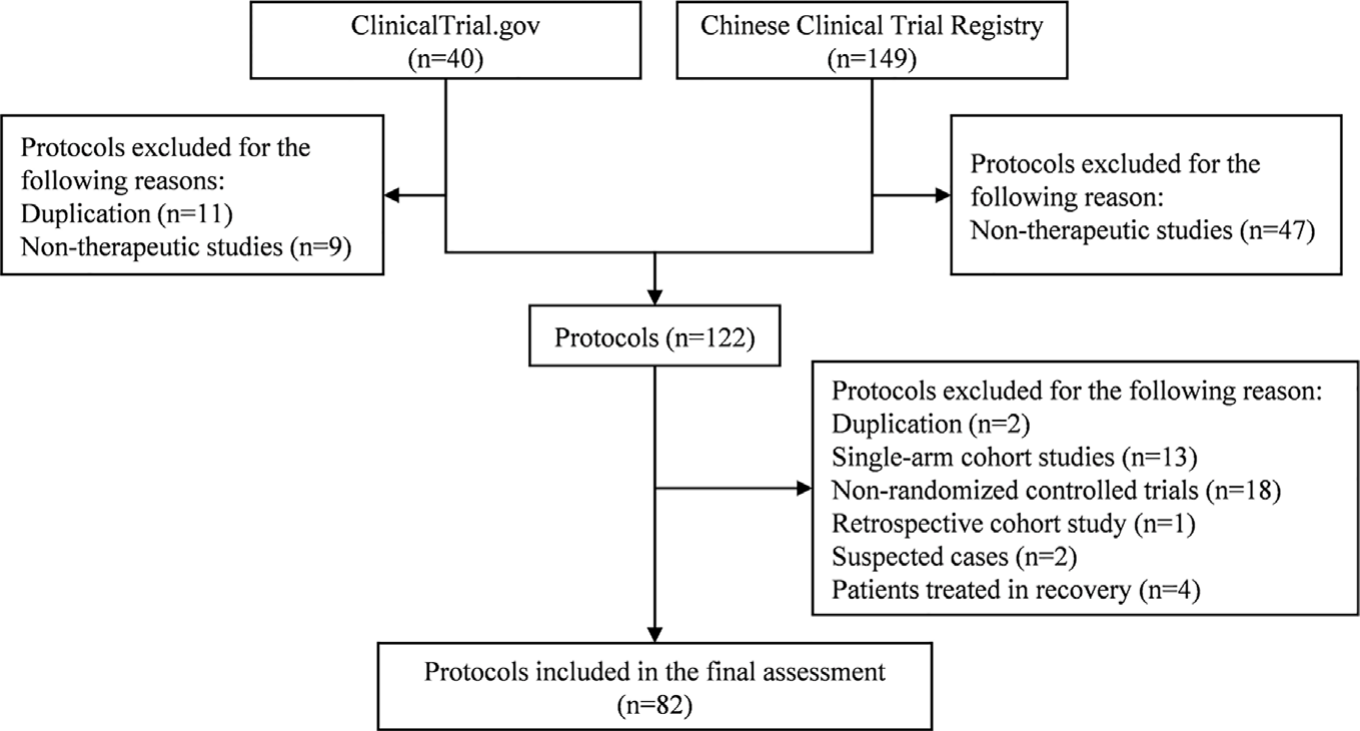
Flow diagram of protocol selection process for this survey.

A total of 189 trial protocols were retrieved from ClinicalTrials.gov and CCTR. After selection (**Figure 1**), we included 82 RCT protocols (17 from ClinicalTrials.gov and 65 from CCTR) in the final assessment. The included trials are being conducted in secondary and tertiary hospitals from 16 provincial areas in China, including Beijing, Shanghai, Chongqing, Hubei, Hunan, Hebei, Henan, Guangdong, Zhejiang, Jiangsu, Shandong, Heilongjiang, Liaoning, Anhui, Shanxi, and Sichuan. The estimated study duration of 51 (62.20%) trials will be longer than six months, except for two trials (ChiCTR2000029762 and ChiCTR2000029855) which did not provide details on the estimated date of completion. Fourteen (17.07%) trials are funded by pharmaceutical companies and 32 (39.02%) trials by the government, while no information about funding source is available for the rest 36 (43.90%) trials.

### Types of Study Participants

Six (7.32%) trials aim to enroll laboratory-confirmed and suspected or clinically diagnosed COVID-19 cases (**Table 1**). Laboratory-confirmed COVID-19 cases could be clinically mild, ordinary, severe, and critical on basis of the classifications of GCVD2019PCDM. Only one (1.22%), nine (10.98%), nine (10.98%), and three (3.66%) trials included mild, ordinary, severe, and critical laboratory-confirmed cases, respectively. The remaining 54 (65.85%) trials plan to recruit more than two subtypes of laboratory-confirmed cases. The participants of 71 (86.58%) trials are adults only and six trials (7.32%) also include children aged 12 years or above, except for five (6.10%) trials without any description of the participant age. Twenty-four (29.27%) trial protocols clearly described the recruitment time (3 to 14 days). Study sample size ranges from 20 to 600, and a total of 32 (39.02%) trials are small-scale studies with less than 100 subjects.

**Table 1:** Characteristics of included trial protocols (n = 82).

No.	Trial registration number	Participants	Participant Age (years)	Sample Size	Treatment comparison	Primary Outcome
1	NCT04252664	Confirmed ordinary cases	≥18	308	Remdesivir Placebo	1
2	NCT04257656^a^	Confirmed severe cases	≥18	452	Remdesivir Placebo	1
3	ChiCTR2000029496^a,b^	Confirmed mild and severe cases	18–70	200	Novaferon + Lopinavir/ritonavir + ST Lopinavir/ritonavir + ST Novaferon + ST ST	2
4	ChiCTR2000029573^c^	Confirmed mild, ordinary, and severe cases	18–66	600	Novaferon + Lopinavir/ritonavir Novaferon + Arbidol Lopinavir/ritonavir Arbidol	2
5	ChiCTR2000029539	Clinically diagnosed and confirmed ordinary cases	≥18	328	Lopinavir/ritonavir + ST ST	3
6	NCT04252885	All confirmed cases	18–80	125	Lopinavir/ritonavir + ST Arbidol + ST ST	2
7	NCT04255017	Confirmed ordinary, severe, and critical cases	≥18	400	Lopinavir/ritonavir + ST Arbidol + ST Oseltamivir + ST ST	1, 4
8	ChiCTR2000029541	All confirmed cases	18–65	100	Lopinavir/ritonavir + Thymosin +ST Darunavir/cobicistat + Thymosin +ST Thymosin +ST	2
9	ChiCTR2000029308	Clinically diagnosed and confirmed severe cases	≥18	160	Lopinavir/ritonavir + Interferon-α2b ST	1, 5
10	ChiCTR2000029387^c^	Confirmed mild cases	≥18	108	Lopinavir/ritonavir + Ribavirin + Interferon-α1b Lopinavir/ritonavir + Interferon-α1b Ribavirin + Interferon-α1b	2
11	NCT04261907	Confirmed mild and ordinary cases	18–75	160	Lopinavir/ritonavir + ST ASC09/ritonavir + ST	3
12	ChiCTR2000029548	Confirmed mild, ordinary, and severe cases	18–75	30	Lopinavir/ritonavir Favipiravir Baloxavir	1, 2
13	ChiCTR2000029741	Confirmed mild and ordinary cases	≥18	112	Lopinavir/ritonavir Chloroquine	2, 3, 6, 7, 8, 9
14	ChiCTR2000029760^d^	All confirmed cases	NA	240	Lopinavir/ritonavir Hydroxychloroquine	1
15	ChiCTR2000029759^d^	Confirmed mild and ordinary cases	18–80	60	Lopinavir/ritonavir + Interferon-α Arbidol + Interferon-α ASC09F+ Interferon-α	1
16	ChiCTR2000029867	All confirmed cases	18–75	520	Lopinavir/ritonavir Carrimycin	1, 2, 5
17	NCT04261270	Confirmed mild and ordinary cases	18–55	60	ASC09F + Oseltamivir Ritonavir + Oseltamivir Oseltamivir	3
18	ChiCTR2000029544	Confirmed mild, ordinary, and severe cases	18–75	30	Favipiravir + Current antiviral treatment Baloxavir + Current antiviral treatment Current antiviral treatment	1, 2
19	ChiCTR2000029939	All confirmed cases	≥18	100	Chloroquine + ST ST	1
20	NCT04261517	All confirmed cases	≥18	30	Hydroxychloroquine + ST ST	2, 6
21	ChiCTR2000029740^a^	All confirmed cases	16–99	200	Hydroxychloroquine ST	2, 4, 8, 9, 10, 12, 13
22	ChiCTR2000029868	Confirmed mild and ordinary cases	≥18	200	Hydroxychloroquine + ST ST	2
23	ChiCTR2000029762^d^	Confirmed severe and critical cases	≥18	60	Hydroxychloroquine + ST ST	2, 4
24	ChiCTR2000029559	All confirmed cases	30–65	300	Hydroxychloroquine (low dose) Hydroxychloroquine (high dose) Placebo	2, 9
25	ChiCTR2000029761^d^	Confirmed ordinary cases	≥18	240	Hydroxychloroquine (low dose) + ST Hydroxychloroquine (medium dose) + ST Hydroxychloroquine (high dose) + ST ST	2, 4
26	NCT04252274	All confirmed cases	NA	30	Darunavir/cobicistat + ST ST	2
27	NCT04260594	Confirmed mild and ordinary cases	18–75	380	Arbidol +ST ST	2
28	NCT04254874	Confirmed ordinary, severe, and critical cases	≥18	100	Interferon (PegIFN-α-2b) + Arbidol +ST Arbidol +ST	1, 4
29	ChiCTR2000029638^a^	Confirmed ordinary, severe, and critical cases	18–75	60	Recombinant super-compound Interferon Interferon-α	2, 4, 5, 9
30	NCT04244591	Confirmed critical cases	≥18	80	Methylprednisolone + ST ST	11
31	ChiCTR2000029656	Confirmed severe cases	≥18	100	Methylprednisolone + ST ST	4, 12, 13, 14
32	ChiCTR2000029386^a,b,e^	Confirmed severe and critical cases	≥18	40	Methylprednisolone + Lopinavir/ritonavir + Interferon-α Lopinavir/ritonavir + Interferon-α	1, 6
33	NCT04263402	Confirmed severe cases	≥18	100	Methylprednisolone (<40mg/d) + ST Methylprednisolone (40-80mg/d) + ST	1, 3
34	NCT04261426	Confirmed severe and critical cases	≥18	80	Intravenous Immunoglobulin ST	1, 11
35	ChiCTR2000029431	All confirmed cases	≥18	45	M1suppression therapy+ Methylprednisolone +ST Methylprednisolone + ST ST	4, 15
36	NCT04268537	Confirmed critical cases	≥18	120	Anti-PD-1 antibody Thymosin + ST ST	11
37	ChiCTR2000029806	Confirmed critical cases	≥18	120	Thymosin Camrelizumab ST	11
38	ChiCTR2000029765	Confirmed ordinary and severe cases	18–85	188	Tocilizumab + ST ST	1
39	ChiCTR2000029974	Confirmed mild and ordinary cases	≥18	300	Probiotics + ST ST	1
40	ChiCTR2000029849	Confirmed severe cases	18–75	60	Regulating intestinal flora + ST ST	6, 7
41	NCT04251767	Confirmed severe cases	14–70	40	Washed microbiota transplantation + ST Placebo + ST	1
42	NCT04264533	Confirmed severe and critical cases	≥18	140	Vitamin C + Water for injection Water for injection	16
43	ChiCTR2000029569	Confirmed severe and critical cases	≥18	30	Umbilical cord mesenchymal stem cell conditioned medium + ST ST	17
44	ChiCTR2000029816	Confirmed mild, ordinary, and severe cases	≥18	60	Cord blood mesenchymal stem cells preparations + ST ST	1
45	ChiCTR2000029606	All confirmed cases	1–99	63	Artificial liver therapy+ Human menstrual blood-derived stem cells preparations + ST Human menstrual blood-derived stem cells preparations + ST Artificial liver therapy + ST ST	6
46	ChiCTR2000029572	Confirmed severe and critical cases	≥18	30	Umbilical cord blood mononuclear cells preparations + ST ST	17
47	ChiCTR2000029812	Confirmed mild, ordinary, and severe cases	≥18	60	Umbilical cord blood mononuclear cells preparations + ST ST	1
48	ChiCTR2000029817	Confirmed mild, ordinary, and severe cases	≥18	60	High-dose NK cells and mesenchymal stem cells Conventional-dose NK cells and mesenchymal stem cells Preventive-dose NK cells and mesenchymal stem cells	1
49	ChiCTR2000029757^a,c^	Confirmed severe cases	≥18	300	Convalescent plasma therapy + ST ST	1
50	ChiCTR2000029818	Confirmed mild, ordinary, and severe cases	≥18	60	Umbilical cord blood plasma preparations + ST ST	1
51	ChiCTR2000029972	Confirmed ordinary, severe, and critical cases	18–65	40	Ultra short wave electrotherapy ST	2, 5
52	ChiCTR2000029768	Confirmed ordinary cases	18–75	60	Diammonium Glycyrrhizinate + Vitamin C + Current antiviral treatment Current antiviral treatment	1
53	ChiCTR2000029776	Confirmed mild and ordinary cases	≥18	40	Polyinosinic-polycytidylic acid injection + ST ST	1
54	ChiCTR2000029811	Confirmed mild, ordinary, and severe cases	≥18	60	Anti-aging active freeze-dried powder granules + ST ST	1
55	ChiCTR2000029851	Confirmed severe and critical cases	35–74	68	Lipoic acid + ST Placebo + ST	18
56	ChiCTR2000029853	Confirmed mild, ordinary, and severe cases	≥18	20	Azvudine ST	2, 3, 4, 5, 6, 7, 8, 9
57	ChiCTR2000029434^a,b,e^	All confirmed cases	≥18	400	Lianhua Qingwen (low dose) + ST Lianhua Qingwen (medium dose) + ST Lianhua Qingwen (high dose) + ST ST	1, 3, 5
58	ChiCTR2000029605	Confirmed ordinary cases	≥18	400	Shuanghuanglian (low dose) + ST Shuanghuanglian (medium dose) + ST Shuanghuanglian (high dose) + ST ST	1
59	ChiCTR2000029742	Clinically diagnosed and confirmed ordinary and Severe cases	18–70	90	Confirmed ordinary cases: (Sodium Aescinate + ST) vs ST Confirmed severe cases: (Sodium Aescinate + ST) vs (Hormonotherapy + ST) vs ST	4
60	ChiCTR2000029755	Confirmed ordinary cases	≥18	120	Jinyebaidu granules + ST ST	Unclear
61	ChiCTR2000029756	All confirmed cases	18–60	238	Xiyanping injection Interferon-α	2, 4, 5, 8, 13, 16
62	ChiCTR2000029780	All confirmed cases	≥18	160	Shenqi Fuzheng injection + ST ST	1
63	ChiCTR2000029781	All confirmed cases	≥18	160	Kangbingdu granules + ST ST	5
64	ChiCTR2000029813	Confirmed mild and ordinary cases	18–75	72	Tanreqing capsules + ST ST	2, 5
65	ChiCTR2000029822	All confirmed cases	NA	110	Honeysuckle decoction Placebo	1
66	ChiCTR2000029954	Clinically diagnosed and all confirmed cases	18–65	300	Honeysuckle oral liquid (low dose) + ST Honeysuckle oral liquid (high dose) + ST ST	1, 17
67	ChiCTR2000029769	Confirmed severe cases	18–80	40	Babaodan + ST ST	9, 10
68	ChiCTR2000029777	Confirmed severe cases	18–80	160	Truncation and Torsion Formula + ST ST	4, 10
69	ChiCTR2000029855	Confirmed ordinary cases	18–75	180	TCM Qingfei prescription + Compound houttuynia mixture TCM Qingfei Prescription WM	2, 5, 19
70	ChiCTR2000029869	Confirmed ordinary, severe, and critical cases	18–80	300	Truncated Torsion’ Formula + ST ST	4, 10
71	ChiCTR2000029941	Suspected cases and confirmed mild, ordinary, and severe cases	18–75	200	TCM + WM WM	3
72	ChiCTR2000029438	Confirmed severe and critical cases	NA	100	TCM + WM WM	7, 16, 17
73	NCT04251871	Confirmed Mild, ordinary, and severe cases	14–80	150	TCM + Oxygen therapy + Interferon-α+ Lopinavir/ritonavir Oxygen therapy + Interferon-α+ Lopinavir/ritonavir	5
74	ChiCTR2000029747	All confirmed cases	12–80	200	TCM WM	4, 9, 19
75	ChiCTR2000029788	All confirmed cases	18–80	60	TCM + WM WM	2, 5, 8, 19
76	ChiCTR2000029790	All confirmed cases	18–80	120	TCM + WM WM	19
77	ChiCTR2000029418	Confirmed severe cases	≥18	42	TCM + WM WM	3
78	ChiCTR2000029439	Confirmed ordinary cases	NA	120	TCM + WM WM	2, 5
79	ChiCTR2000029461	Confirmed ordinary cases	18–70	100	TCM + WM WM	2, 5, 11
80	ChiCTR2000029518	Confirmed ordinary and severe cases	14–80	140	TCM + WM WM	1, 3
81	ChiCTR2000029763	Confirmed ordinary cases	18–75	408	TCM + ST ST	3
82	ChiCTR2000029601	Suspected cases and confirmed ordinary cases	18–65	400	TCM + WM + Health education WM + Health education	2, 3, 20

^a^The updated protocol adjusted the sample size; ^b^The updated protocol changed the intervention and comparison groups; ^c^The updated protocol changed the inclusion and exclusion criteria of participants; ^d^The protocol was withdrawn; ^e^The updated protocol changed the primary outcomes; NA, Not Available; ST, Standard Treatment; TCM, Traditional Chinese Medicine; WM, Western Medicine; 1, Rate of or time to disease remission or recovery; 2: Rate of or time to virus-negative conversion; 3: Rate of or time to composite adverse outcome; 4: Rate of or time to lung imaging recovery; 5: Rate of or time to clinical symptom remission; 6: All-cause mortality or mortality; 7: Length of hospitalization; 8: Oxygenation index; 9: Results of routine laboratory tests; 10: Prognosis of patients; 11: Lower Murray lung injury score; 12: Incidence of complications; 13: Vital physiologic parameters; 14: National Early Warning Score (NEWS) 2; 15: Computed Tomography (CT) and Magnetic Resonance Imaging (MRI) of hip; 16: Rate of mechanical ventilation support or ventilation-free days; 17: Pneumonia Severity Index; 18: Lower Sequential Organ Failure Assessment (SOFA) score; 19: TCM symptom score; 20: Confirmed rate of suspected cases.

### Types of Study Interventions and Comparators

The majority of the included trials (n = 56; 68.29%) use WM as the study intervention, with the remaining 26 (31.71%) trials evaluating the effects of TCM. For the former, interventions include interferon aerosol inhalation, lopinavir/ritonavir, ribavirin, chloroquine phosphate, arbidol, and remdesivir, with lopinavir/ritonavir being the most common study intervention (n = 13; 15.85%). The interventions of TCM are more diverse, including Lianhua Qingwen, Shuanghuanglian, Aescinate, Jinyebaidu granule, Xiyanping injection, Shenqi Fuzheng injection, Kangbingdu granule, Tanreqing capsule Honeysuckle decoction, etc.

### Types of Primary Outcomes

We found one trial protocol (ChiCTR2000029755) without specifying a primary outcome measure. Seven (8.54%) protocols included more than three primary outcomes but none set any primary outcomes regarding safety. We obtained 20 primary outcomes from the 82 protocols assessed and classified them into six groups: (1) the prognostic outcome [rate of or time to disease remission or recovery, rate of or time to composite adverse outcome, all-cause mortality or mortality, length of hospitalization, patient prognosis, complication incidence, National Early Warning Score (NEWS) 2, Pneumonia Severity Index (PSI), and Sequential Organ Failure Assessment (SOFA) score]; (2) the etiological outcomes (rate of or time to virus-negative conversion of SARS-CoV-2); (3) outcomes on clinical symptoms (rate of or time to no fever, no cough, no dyspnea, or no myalgia); (4) outcomes about the lung or respiratory function (e.g., rate of or time to lung imaging recovery, lung injury score, oxygenation index, requirements of mechanical ventilation support, etc.); (5) outcome assessed using the TCM symptom score; (6) outcome about the vital physiologic parameters (e.g., body temperature, blood pressure, heart rate, and breathing rate) and routine laboratory tests (e.g., routine blood test, C-reaction protein, procalcitonin, creatine kinase, alanine aminotransferase, CD4, CD8, interleukin, etc.).

### Quality Assessment by SPIRIT 2013 Statement

**Figure 2:**
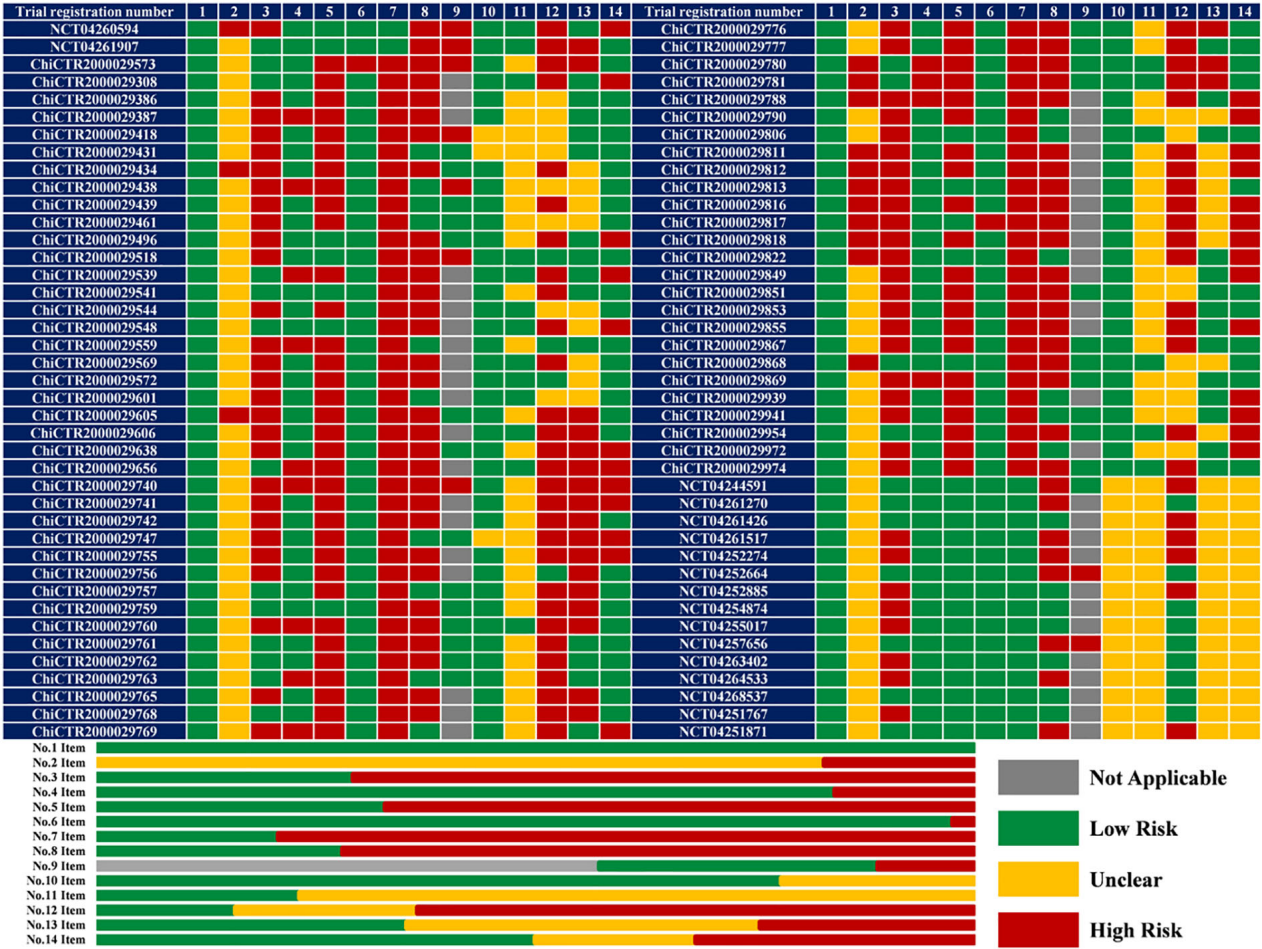
The results of quality assessment (n = 82). 1 or No.1 Item: Specific objectives or hypotheses; 2 or No.2 Item: conflict of interest; 3 or No.3 Item: clear enrolment schedule; 4 or No.4 Item: specific participant inclusion and exclusion criteria; 5 or No.5 Item: sufficient details about interventions for each group; 6 or No.6 Item: matching between grouping and the research purpose; 7 or No.7 Item: sufficient details about outcome measurement; 8 or No.8 Item: suitability of the primary outcome; 9 or No.9 Item: all the collaborating institutions listed in a multicenter study; 10 or No.10 Item: randomization sequence generation; 11 or No.11 Item: allocation concealment; 12 or No.12 Item: blinding; 13 or No.13 Item: data collection and management methods; 14 or No.14 Item: ethical permit.

Limitations in terms of methodology existed across all the included protocols (**Figure 2**). Although the quality of protocols registered on the ClinicalTrial.gov was better than those registered the on CCTR, their assessment results about five items (No. 2, 10, 11, 13, and 14) could not been performed due to unavailable information regarding funding resource, ethics materials, methods of random sequence generation, allocation concealment, data collection, and management.

All included protocols clearly described study objectives. Fifty-six (68.29%) trials investigate both efficacy and safety endpoints while the remaining (31.71%) 26 trials focus only on treatment efficacy. The potential risk of bias due to conflict of interest was noted in 14 (17.07%) trials sponsored by the pharmaceutical companies. A recruitment time of 58 (70.73%) trials was not mentioned, which would cause a high risk of attrition and reporting bias. The inclusion and exclusion criteria of 16 (19.51%) protocols were ambiguous due to undescribed participant age (n = 5), inconsistency between the participants and the study purpose (n = 4), incorrect clinical classifications (n = 4), and absence of overall detailed criteria (n = 3). Fifty-five (67.07%) protocols did not describe the intervention and comparison in detail, particularly regarding the course of treatment, and the selection of control group in two protocols (ChiCTR2000029573 and ChiCTR2000029817) could not match their study purpose. Sixty-five (79.27%) protocols did not define the outcomes, especially in detection timepoint.

The primary outcomes of 59 (71.95%) protocols were inappropriate: excessive number of primary outcomes for 7 trials; no safety-related endpoints as primary outcomes for 56 trials evaluating safety (39 did not specify any safety-related outcomes). Among 35 multicenter trials, 9 protocols did not list all the collaborating medical institutions. Despite the accurate methods of random sequence generation (random number table or computer-generated random numbers) in 64 (78.05%) protocols, descriptions of allocation concealment in 63 (76.83%) protocols remained unclear. Therefore, the overall risk of selection bias across these RCTs was unclear. Masking methods in 13 (15.85%) trials varied, from single-blind (participant or outcomes assessor; n = 7), double-blind (participant and care provider; n = 2), triple-blind (participant, care provider and outcomes assessor; n = 1), to quadruple-blind (participant care provider, investigator, and outcomes assessor; n = 3). Nevertheless, 52 (63.41%) trials are of open-label design which is associated with a high risk of performance bias and detection bias. Twenty-nine (35.36%) trials have the Data Management Committee but 20 (24.39%) trials do not. The ethics materials of 26 (31.71%) protocols were incomplete: 11 protocols were not approved by the Ethics Committee, 6 protocols were without available approved file, and 9 protocols did not mention the informed consensus.

## Discussion

This survey assessed the quality of 82 RCT protocols regarding treatments for COVID-19 in 16 provincial areas in China. We found that: (1) The study duration of most trials (62.20%) is more than six months which might be too long to enroll enough participants as the COVID-19 will be gradually controlled. Recently, 4 protocols (ChiCTR2000029760, ChiCTR2000029759, ChiCTR2000029762, and ChiCTR2000029761) were withdrawn due to inadequate numbers of patients. (2) These trials mainly focus on COVID-19 mild, ordinary, and severe cases, and the number of trials for WM is more than that for TCM. (3) Eighty-two protocols set 20 different primary outcomes, indicating considerable controversy in the primary outcome for evaluating the efficacy of COVID-19 treatments. Furthermore, only 17 trials (20.73%) considered the safety profile of therapies. (4) The protocols should be improved from several aspects, such as the selection and definition of outcomes, intervention and comparison, recruitment time, inclusion and exclusion criteria, and allocation concealment method. In addition, it is necessary to standardize basic treatments and select appropriate outcomes to reduce the high risk of performance and detection bias in the context of a non-blind design.

Participants of the 82 included trials are suspected, clinically diagnosed, or laboratory-confirmed cases; the majority (73.17%) of these trials recruit more than two subtypes of laboratory-confirmed cases. Given the differences in clinical characteristics, basic treatments, and prognosis of different subtypes, subgroup analyses are strongly suggested in evaluating the efficacy of study interventions. Nevertheless, the sample size of 26 trials are less than 100 and another 4 updated protocols (ChiCTR2000029496, ChiCTR2000029740, ChiCTR2000029757, and ChiCTR2000029434) greatly cut down their sample size, which might compromise the power of statistical analysis. It is critical to detail interventions for future replication study; however, most protocols (67.07%) did not provide any information about the course of treatment, particularly those related to TCM.

One utmost prerequisite to evaluate the treatments for COVID-19 is to determine appropriate outcomes. First of all, the outcomes related to efficacy and safety profiles are of equal importance for a new type of intervention, whereas 26 protocols focused on efficacy only. Even for those trials designed to evaluate the safety profile, only a few selected the incidence of adverse events or severe adverse events as the secondary outcome. Secondly, the primary outcome should represent the greatest therapeutic benefit and be the most important among the many outcomes (Sedgwick, 2010; Andrade, 2015). However, 9 (10.98%) protocols set intermediate outcomes (vital physiologic parameters or routine laboratory tests) as the primary outcome and 7 (8.54%) protocols adopted more than three primary outcomes. Thirdly, the primary outcomes should be generally similar for different protocols with the same study purpose. Nevertheless, there is a variety of primary outcomes among the present protocols regarding investigational interventions for COVID-19. We noted that the time to clinical improvement within 28 days, the lethality by day 28, the rate of symptom (fever, fatigue, and coughing) recovery, the time to achieve a negative RT-PCR result for SARS-CoV-2 in a nasopharyngeal swab sample were set as the primary outcomes in trials regarding remdesivir (Beigel et al., 2020; Wang Y. et al., 2020), chloroquine diphosphate (Borba et al., 2020), Lianhuaqingwen capsules (Hu et al., 2020), and triple combination of interferon beta-1b, lopinavir/ritonavir, and ribavirin (Hung et al., 2020), respectively. In addition, Wang Y et al. (Wang Y. et al., 2020), Cao B et al. (Cao et al., 2020), and Beigel JH et al. (Beigel et al., 2020) used different tools (six-point, seven-category, and eight-category ordinal scale) to measure clinical improvement or recovery which, in effect, was proposed as the most critical endpoint by other trial investigators. The significant heterogeneity and lack of critical outcomes across these COVID-19 studies may lead to a waste of research resources.

We argue that the selection of primary outcome should be based on expert consensus and/or conventional practices. For instance, mortality can be the primary outcome for laboratory-confirmed critical cases. Importantly, clinical classifications of participants should be considered while using mortality as the primary outcomes since laboratory-confirmed mild or ordinary cases have a better prognosis. As to the laboratory-confirmed mild cases, the rate of or the time to disease recovery might be a better primary outcome compared to lung imaging recovery. For the laboratory-confirmed ordinary or severe cases, two optional primary outcomes could be the rate of/the time to disease remission (improvement from severe cases to ordinary cases) and the rate of/the time to composite adverse outcome (admission to an intensive care unit, the use of mechanical ventilation, or death). Due to the high rate of false-negative results of the nucleic acid test of 2019-nCoV, etiological outcome is not suggested as the unique primary outcome despite its specificity. In fact, 9 (10.98%) protocols selected the etiological outcome as the only primary outcome. Additionally, the results of the nucleic acid test of SARS-CoV-2 is an inappropriate outcome for suspected and clinically diagnosed cases. Although TCM symptom score was adopted as the primary outcome in four (4.88%) protocols, it is still controversial in TCM-relevant studies. A recent study (Jin et al., 2020) demonstrated a core outcome set of different outcome measures for different subtypes of laboratory-confirmed COVID-19 cases based on two rounds of Delphi survey and one consensus meeting, and we are confident that such efforts to develop core outcome sets would be useful for future evidence synthesis and clinical decision-making.

Furthermore, only 17 (20.73%) protocols clearly described how to measure the primary outcomes but the detection time varied a lot. Most protocols agreed that the first week after treatment is important for laboratory-confirmed severe or critical cases and the second week after treatment is critical to evaluate the outcomes regarding the prognosis of COVID-19. A few protocols suggested a longer time (4 weeks or even longer) for mortality measurement. The first week after treatment was also proposed by most protocols for measuring the etiological outcome, while Chen J et al. (Chen J. et al., 2020) considered weeks necessary to detect the rate of virus-negative conversion.

With the spread of COVID-19, increasing clinical trials will be initiated to evaluate the efficacy and safety of potential therapies. The protocol determines the quality of study methodology and the reliability of conclusion, and is thus fundamental to the design, implementation, report, and assessment of a clinical trial.

A previous study investigated 172 trial protocols regarding COVID-19 and found issues related to necessity, scientific validity, ethics, and quality (Xiang et al., 2020). Another cross-sectional analysis characterized trial intervention, sponsorship, critical design elements, and specified outcomes of 201 clinical trials assessing drugs or plasma treatments for COVID-19 and concluded that many trials lacked features to optimize their scientific value (Mehta et al., 2020). Unfortunately, they did not assess the methodological quality of these protocols. The 33-item SPIRIT Statement is a powerful tool for assessing the quality of published protocols; however, it does not apply to protocols registered on ClinicalTrials.gov and the CCTR, which often contain incomplete information. Consequently, we modified the original SPIRIT 2013 Statement into a more concise 14-item checklist for preliminary assessment of the methodological quality of trial protocols regarding treatments for COVID-19. In the context of the absence of tool for assessing the quality of registry protocols, our study provides a paradigm for future assessments and also might guide study design of clinical trial. The limitations of this study must be acknowledged. All the included trial protocols were from China and 31.71% were related to TCM, which may reduce the generalizability of our results to clinical trial investigators from around world. Furthermore, we were unable to judge whether the statistical power is sufficient in the absence of information about sample estimation and statistical methods. It is worth highlighting that a recent trial of lopinavir/ritonavir in COVID-19 was statistically underpowered and the findings/conclusions indicating that lopinavir/ritonavir was ineffective for COVID-19 patients should thus be interpreted with caution (Carmona-Bayonas et al., 2020). Therefore, further assessment of the trials in terms of methodological quality will be performed after the trials are completed. In fact, the WHO, NIH, etc., have suspended trials/part trials (WHO Solidarity trial and UK Recovery Trial) with an arm of chloroquine, hydroxychloroquine, or lopinavir/ritonavir due to no benefitial effect of these antivirals in patients with COVID-19 (Borba et al., 2020; Boulware et al., 2020; Carmona-Bayonas et al., 2020; Griffin, 2020; Mitja et al., 2020; Skipper et al., 2020; Tang et al., 2020; Torjesen, 2020). In China, four relevant trials (ChiCTR2000029760, ChiCTR2000029759, ChiCTR2000029762, ChiCTR2000029761) have also been suspended. We will continuously follow the progress of these trials and also appeal to improvements of registry protocols in line with SPIRIT 2013.

## Conclusion

Currently, available RCT protocols on potential therapies for CoVID-19 have significant methodological limitations, especially in selection and detection of primary outcomes. Further assessment of trial quality should be performed after the completion of those trials. If the trials are not designed with strict standards, the effort will be in vain. Therefore, despite eager anticipation from the public on the results of COVID-19 therapeutic trials, we must maintain cautious and rigorous on the trial design.

## Data Availability Statement

Publicly available datasets were analyzed in this study. This data can be found here: The datasets generated for this study are available on request to the corresponding author.

## Author Contributions

JZ, YL, and XL collected the data. JZ and XL involved in statistical analysis. JZ, YL, and XL drafted the manuscript. JZ, JS-WK, WZ, and RH revised the final manuscript. All authors contributed to the article and approved the submitted version.

## Conflict of Interest

The authors declare that the research was conducted in the absence of any commercial or financial relationships that could be construed as a potential conflict of interest.

The handling editor declared a shared affiliation, though no other collaboration, with two of the authors WZ, RH.
